# Supplementary material for: Being Underweight Is Associated with Increased Risk of Sudden Cardiac Death in People with Diabetes Mellitus
Source: J Clin Med. 2023 Jan 29;12(3):1045. doi: 10.3390/jcm12031045 (PMC9917578; doi:10.3390/jcm12031045)
Supplement: Supplementary file 1 [file jcm-12-01045-s001.zip › jcm-2166420-supplementary.pdf]

Being Underweight is Associated with Increased Risk of Sudden Cardiac Death in People with Diabetes Mellitus

Yun Gi Kim[1], Kyung-Do Han[2], Seung-Young Roh[3], Joo Hee Jeong[1], Yun Young Choi, Kyongjin Min[4], Jaemin Shim[1], Jong-Il Choi[1]*, and Young-Hoon Kim[1]

[1]Division of Cardiology, Department of Internal Medicine, Korea University College of Medicine and Korea University Anam Hospital, Seoul, Republic of Korea

[2]Department of Statistics and Actuarial Science, Soongsil University, Seoul, Republic of Korea

[3]Division of Cardiology, Department of Internal Medicine, Korea University College of Medicine and Korea University Guro Hospital, Seoul, Republic of Korea

[4]Division of Cardiology, Department of Internal Medicine, Sangye Paik Hospital, Inje University College of Medicine, Seoul, Republic of Korea

*Address for correspondence: Jong-Il Choi, MD, PhD, MHSc

Division of Cardiology, Department of Internal Medicine, Korea University College of Medicine and Korea University Anam Hospital, Seoul, Republic of Korea

73 Goryeodae-ro, Seongbuk-gu, Seoul 02841, Republic of Korea

Tel: 82-2-920-5445

Fax: 82-2-927-1478

E-mail: jongilchoi@korea.ac.kr

Running title: Underweight paradox in diabetes

Supplementary Figure S1. Cumulative incidence of SCD.

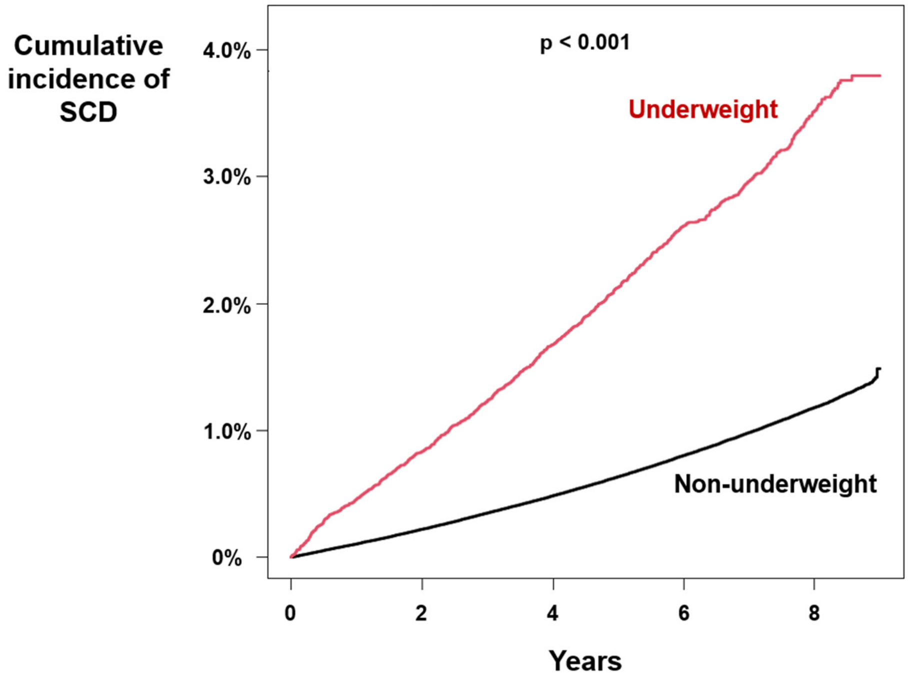

Kaplan-Meier curve analysis revealed a significantly increased risk of SCD in DM people who were underweight.

Underweight: BMI < 18.5 kg/m^2; non-underweight: BMI ≥ 18.5 kg/m^2

DM: diabetes mellitus; SCD: sudden cardiac death.

Supplementary Table S1. Risk of SCD according to BMI value (people and event numbers).

BMI	n	SCD	Follow-up duration (person-years)	Incidence	Hazard ratio with 95% confidence interval		
					Non-adjusted	Model 1	Model 2
<17	9,913	358	53,595	6.68	4.611 (4.134 – 5.143)	3.656 (3.277 – 4.079)	3.491 (3.127 – 3.896)
17	16,483	401	98,981	4.05	2.761 (2.489 – 3.063)	2.477 (2.233 – 2.748)	2.377 (2.141 – 2.638)
18	36,361	730	228,178	3.20	2.169 (2.001 – 2.350)	2.058 (1.899 – 2.231)	1.969 (1.816 – 2.134)
19	69,504	1,205	450,241	2.68	1.806 (1.690 – 1.930)	1.744 (1.633 – 1.864)	1.681 (1.572 – 1.796)
20	117,241	1,690	777,155	2.17	1.462 (1.378 – 1.551)	1.431 (1.349 – 1.517)	1.393 (1.313 – 1.478)
21	186,851	2,360	1,261,789	1.87	1.254 (1.189 – 1.322)	1.231 (1.167 – 1.298)	1.208 (1.145 – 1.274)
22	253,451	2,829	1,733,092	1.63	1.093 (1.039 – 1.149)	1.078 (1.025 – 1.134)	1.066 (1.014 – 1.122)
23	312,269	3,217	2,150,866	1.50	1 (reference)	1 (reference)	1 (reference)
24	333,175	3,184	2,306,935	1.38	0.922 (0.878 – 0.969)	0.933 (0.889 – 0.980)	0.941 (0.896 – 0.988)
25	318,170	2,831	2,209,341	1.28	0.856 (0.814 – 0.900)	0.895 (0.851 – 0.941)	0.904 (0.859 – 0.951)
26	276,135	2,278	1,919,880	1.19	0.793 (0.751 – 0.837)	0.859 (0.814 – 0.906)	0.868 (0.822 – 0.916)
27	214,291	1,694	1,489,636	1.14	0.760 (0.717 – 0.806)	0.858 (0.809 – 0.910)	0.864 (0.815 – 0.917)
28	151,407	1,204	1,051,794	1.14	0.766 (0.717 – 0.818)	0.924 (0.864 – 0.987)	0.925 (0.865 – 0.988)
29	106,436	837	737,927	1.13	0.760 (0.704 – 0.820)	0.968 (0.897 – 1.044)	0.967 (0.896 – 1.043)
30	73,563	526	508,753	1.03	0.693 (0.632 – 0.760)	0.954 (0.870 – 1.046)	0.944 (0.860 – 1.035)
31	41,914	323	288,861	1.12	0.750 (0.669 – 0.841)	1.117 (0.996 – 1.252)	1.092 (0.973 – 1.225)
32	32,389	266	223,394	1.19	0.799 (0.705 – 0.905)	1.265 (1.116 – 1.434)	1.228 (1.083 – 1.392)
33	17,489	131	119,701	1.09	0.736 (0.618 – 0.877)	1.262 (1.059 – 1.503)	1.219 (1.023 – 1.452)
34	12,259	91	83,966	1.08	0.729 (0.592 – 0.898)	1.358 (1.103 – 1.674)	1.300 (1.055 – 1.602)
≥35	23,276	186	157,710	1.18	0.796 (0.687 – 0.923)	1.702 (1.467 – 1.974)	1.584 (1.365 – 1.838)

Incidence is per 1,000 person-years of follow-up.

BMI: body-mass index; SCD: sudden cardiac death.

Model 1: adjusted for age and sex

Model 2: adjusted for age, sex, income level, smoking history, alcohol consumption, regular physical activity, hypertension, dyslipidemia, fasting glucose, duration of diabetes mellitus, use of insulin, and number of oral antidiabetic medications.

Supplementary Table S2. Subgroup analysis (people and event numbers).

		Underweight	n	SCD	Person*year	Incidence	Adjusted-HR (95% CI)	p for interaction
Age groups	< 40	No	191,481	310	1,336,012	0.23	1 (reference)	< 0.001
		Yes	5,172	12	35,739	0.34	2.11 (1.18 – 3.75)	
	40 ≤ age < 64	No	1,601,863	10,444	11,198,048	0.93	1 (reference)	
		Yes	18,663	447	119,626	3.74	3.36 (3.06 – 3.70)	
	≥ 65	No	767,635	14,505	5,070,664	2.86	1 (reference)	
		Yes	17,763	623	91,708	6.79	2.00 (1.84 – 2.17)	
Sex	Male	No	1,538,533	17,788	10,502,864	1.69	1 (reference)	0.660
		Yes	23,765	776	137,824	5.63	2.42 (2.25 – 2.60)	
	Female	No	1,022,446	7,471	7,101,860	1.05	1 (reference)	
		Yes	17,833	306	109,249	2.80	2.35 (2.09 – 2.63)	
Income	Q2-Q4	No	2,024,902	19,554	13,958,317	1.40	1 (reference)	0.245
		Yes	31,221	798	185,743	4.30	2.35 (2.19 – 2.52)	
	Lowest Q1	No	536,077	5,705	3,646,406	1.56	1 (reference)	
		Yes	10,377	284	61,330	4.63	2.55 (2.26 – 2.87)	
Smoking	Non- and Ex-smoker	No	1,901,877	17,859	13,122,128	1.36	1 (reference)	0.513
		Yes	27,451	650	162,541	4.00	2.44 (2.25 – 2.64)	
	Current-smoker	No	659,102	7,400	4,482,596	1.65	1 (reference)	
		Yes	14,147	432	84,532	5.11	2.34 (2.12 – 2.58)	
Drinking	Non- and Mild-drinker	No	2,305,701	22,855	15,854,579	1.44	1 (reference)	0.010
		Yes	37,890	955	225,110	4.24	2.48 (2.33 – 2.65)	
	Heavy-drinker	No	255,278	2,404	1,750,145	1.37	1 (reference)	
		Yes	3,708	127	21,963	5.78	3.19 (2.67 – 3.82)	
Regular exercise	No	No	2,031,019	20,349	13,901,326	1.46	1 (reference)	0.961
		Yes	35,533	939	208,941	4.49	2.40 (2.25 – 2.56)	
	Yes	No	529,960	4,910	3,703,398	1.33	1 (reference)	
		Yes	6,065	143	38,132	3.75	2.39 (2.02 – 2.82)	
Hypertension	No	No	1,098,897	6,989	7,627,811	0.92	1 (reference)	0.087
		Yes	25,633	523	159,360	3.28	2.54 (2.32 – 2.78)	
	Yes	No	1,462,082	18,270	9,976,913	1.83	1 (reference)	
		Yes	15,965	559	87,713	6.37	2.28 (2.10 – 2.49)	
Dyslipidemia	No	No	1,478,761	14,265	10,147,830	1.41	1 (reference)	0.015
		Yes	32,506	850	192,531	4.41	2.50 (2.33 – 2.68)	
	Yes	No	1,082,218	10,994	7,456,894	1.47	1 (reference)	
		Yes	9,092	232	54,542	4.25	2.09 (1.83 – 2.38)	

Insulin	No	No	2,339,021	20,318	16,155,803	1.26	1 (reference)	0.031
		Yes	35,615	839	215,166	3.90	2.49 (2.32 – 2.67)	
	Yes	No	221,958	4,941	1,448,921	3.41	1 (reference)	
		Yes	5,983	243	31,907	7.62	2.12 (1.86 – 2.41)	
Number of oral antidiabetics	< 3	No	2,189,816	20,023	15,026,423	1.33	1 (reference)	0.097
		Yes	35,454	869	211,136	4.12	2.46 (2.30 – 2.64)	
	≥ 3	No	371,163	5,236	2,578,301	2.03	1 (reference)	
		Yes	6,144	213	35,937	5.93	2.16 (1.89 – 2.48)	
DM Duration	< 5 years	No	1,764,270	13,137	12,137,057	1.08	1 (reference)	0.352
		Yes	28,792	628	173,735	3.61	2.46 (2.27 – 2.67)	
	≥ 5 years	No	796,709	12,122	5,467,667	2.22	1 (reference)	
		Yes	12,806	454	73,338	6.19	2.32 (2.11 – 2.55)	

Incidence is per 1,000 person-years of follow-up.

BMI: body-mass index; CI: confidence interval; DM: diabetes mellitus; HR: hazard ratio; SCD: sudden cardiac death.

HRs are adjusted for age, sex, income level, smoking history, alcohol consumption, regular physical activity, hypertension, dyslipidemia, fasting glucose, duration of diabetes mellitus, use of insulin, and number of oral antidiabetic medications.

Q1 represents the lowest income and Q4 represents the highest.
